# High Tumor Mutational Burden in Hepatocellular Carcinoma

**DOI:** 10.7759/cureus.68132

**Published:** 2024-08-29

**Authors:** Joshua A Engle, James T Dibb, John A Jakob

**Affiliations:** 1 Department of Medicine, Summa Health, Akron, USA; 2 Department of Medical Oncology, Summa Health, Akron, USA

**Keywords:** tumor mutational burden, cancer genetics, prognosis, hepatocellular carcinoma, immunotherapy

## Abstract

Hepatocellular carcinoma (HCC) is the most common type of liver cancer and one of the leading causes of cancer-related deaths worldwide. Tumor mutational burden (TMB) in a cancer specimen represents the number of mutations within a pre-determined length of genetic material. This value is increasingly investigated as it may correlate with both prognosis and cancer response to developing immunotherapies. Its role in HCC, however, is still unclear as it tends to exist in a lower range.

We present the case of a 72-year-old female diagnosed with HCC that was diffusely spread throughout the liver without evidence of metastatic disease. Genetic analysis of a liver biopsy revealed a TMB of 87 mutations per megabase which is extremely high for HCC. The patient was treated with tremelimumab and durvalumab but passed away shortly afterward due to decompensation from her disease.

This case highlights the importance of continuing to research TMB and its correlation with this cancer. An HCC case with an exceptionally high TMB that progressed rapidly, like this one, indicates the continued need to study markers such as TMB, especially in outlier cases, for prognostication and appropriate stratification of immunotherapy response.

This case shows an instance of a non-metastatic but still aggressive HCC that was diagnosed too late for assessing therapy response. In this instance, the high TMB would point toward a worse prognosis, but further study of high-TMB cases is necessary to support a correlation. Given the prevalence of HCC worldwide, any potential avenues that could help guide clinical decision-making should be explored.

## Introduction

Hepatocellular carcinoma (HCC) is the most prevalent histologic type of liver cancer and accounts for the majority of liver cancer deaths worldwide. Hepatitis B and hepatitis C are the most important global risk factors for HCC, but metabolic risk factors, such as non-alcoholic fatty liver disease, are present as well [1]. HCC is typically diagnosed later in its course contributing to its poor overall prognosis, with the two-year survival rate in the United States being less than 50% [2].

Treatment options for HCC include hepatectomy depending on tumor location/composition and transarterial chemoembolization. Newer treatments include immunotherapies such as durvalumab, which blocks programmed death ligand 1 (PD-L1), and tremelimumab, which blocks cytotoxic T lymphocyte antigen-4 (CTLA-4) [3]. Predicting cancer response to such therapies is an increasingly researched topic as more treatments are developed. Measurements of such values as tumor mutational burden (TMB) [4], microsatellite instability (MSI), and PD-L1 [5] are being assessed for potential implications in prognosis and choice of therapeutic interventions.

TMB is the total number of somatic non-synonymous mutations per coding area of a tumor genome. It is measured in mutations per megabase (mut/Mb) [6]. Higher numbers of mutations, in theory, lead to an increase in immunogenic recognition and T-cell response. Thus, TMB may determine the overall immunotherapy response in some cancers [7]. Given its potential role in treatment response, it is also evaluated for the overall role in prognosis among different malignancies as well. TMB’s role in determining treatment efficacy and overall prognosis in HCC remains uncertain, especially with the normally lower range and the difficulty in establishing “low” and “high” spectrums [8]. An analysis of 165 HCC patients found TMB values to possess a median of 5.4 mut/Mb with 95% less than 10 mut/Mb and a highest recorded value of 28.4 mut/Mb [9].

With this in mind, we report the case of a 72-year-old female who was diagnosed with a clinically aggressive but non-metastatic HCC with a very high TMB compared to the normal range.

This article was previously presented as a poster at the ACP 2023 Ohio/Air Force Chapters Annual Scientific Meeting on October 20, 2023.

## Case presentation

**Figure 1:**
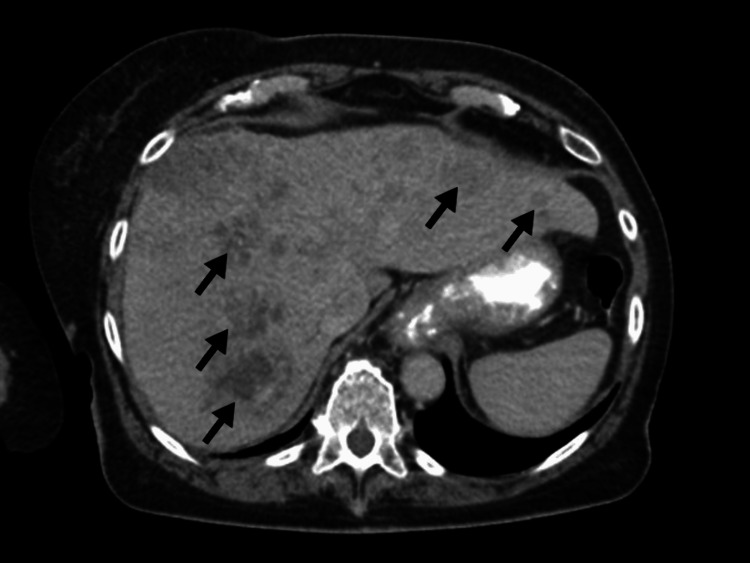
Computed tomography of the abdomen with contrast showing predominantly hypodense masses scattered throughout the liver (arrows).

**Figure 2:**
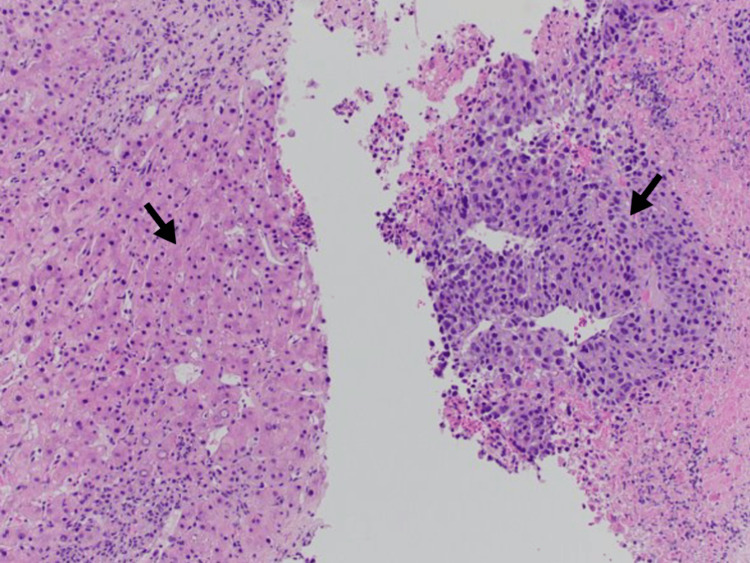
Tissue slide prepared from ultrasound-guided percutaneous liver biopsy with normal tissue (left arrow) and tumor (right arrow) present.

**Figure 3:**
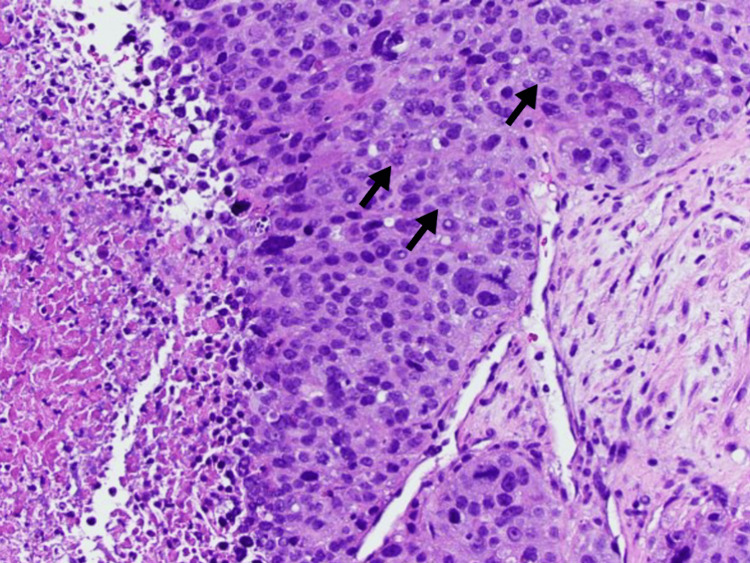
Liver biopsy slide showing malignant cells (arrows) noted to have marked anisonucleosis and numerous mitoses.

A 72-year-old female was seen by her primary care doctor for complaints of new-onset pain in the right upper rib cage and weight loss. Computed tomography (CT) of the abdomen and pelvis revealed a diffusely heterogeneous liver with innumerable hypodense masses with concern for metastatic disease (Figure 1). CT of the chest did not show further evidence of metastatic spread to the chest. Ultrasound-guided percutaneous liver biopsy was performed and tissue slides were prepared (Figure 2). Immunohistochemistry testing was negative for being MSI-high. Gross pathology demonstrated poorly differentiated carcinoma with necrosis. The malignant cells showed marked anisonucleosis and numerous mitoses (Figure 3). Cells stained positive for cytokeratin 20 (CK20) and focally positive for cytokeratin 7 (CK7). Stains for thyroid transcription factor-1 (TTF-1), caudal-type homeobox 2 (CDX2), arginase, hepatocyte paraffin 1 (HepPar1), paired box 8 (Pax8), and GATA3 binding protein (GATA3) were negative. This pattern was non-specific but suspicious for HCC or biliary tract neoplasm. Cholangiocarcinoma profiling was ordered along with alpha-fetoprotein (AFP). AFP was 89,000 ng/mL, with the normal range being less than 6.1 ng/mL, indicating probable stage II HCC (cT2N0M0) with an estimated Child-Pugh class of B7. Profiling analysis detected PD-L1 expression and was negative for fibroblast growth factor receptor 2 (FGFR2) gene rearrangement. TMB of the biopsy resulted at 87 mut/Mb, considered high for HCC which normally has TMB values less than 10 mut/Mb [9]. Palliative therapy was initiated with one dose of tremelimumab and durvalumab every four weeks for 10 cycles. Surgical intervention was not considered as initial treatment due to diffuse infiltration of the hepatic parenchyma. After receiving her first cycle of immunotherapy, she rapidly declined and then died due to hepatic decompensation caused by tumor burden.

## Discussion

HCC significantly contributes to the global cancer burden, and incidence rates have increased in recent decades [1]. As such, it is beneficial to determine reliable markers of immunotherapy response and prognosis. TMB’s role remains unclear but cases such as the one presented may point to its importance. Based on previous studies, most HCC TMB values tend to be less than 10 mut/Mb [9]. This lower observed range is one of the reasons why TMB is challenging to study in HCC, being associated with the difficulty of establishing the lower and higher ends of the value spectrum [8]. Regardless, a TMB of 87 mut/MB, as was seen in this case, is uncommonly high for HCC. After a thorough literature review, no specific mention of a TMB as high as 87 mut/MB could be found for this type of cancer.

One of TMB’s most important clinical roles is the potential utility in predicting response to immunotherapies as they are developed [3]. It has been studied in a multitude of different cancers with various outcomes. High levels of TMB have been shown to correlate with increased responsiveness of non-small-cell lung cancer to immune checkpoint inhibitors nivolumab and ipilimumab [10] and of malignant melanoma to anti-CTLA-4 antibody treatment [11]. The pursuit of identifying similar relationships between TMB and HCC is ongoing. Unfortunately, the patient in this case declined before the efficacy of treatment could be assessed. Only one cycle of immunotherapy was completed before she was hospitalized and ultimately passed away. Despite this outcome, the case highlights the continued need to evaluate the potential TMB range for HCC. Determining potential correlation with responses to immunotherapies such as durvalumab and tremelimumab would then help guide clinical decision-making between provider and patient when treatment options are being discussed.

TMB is often studied for correlation with prognosis in different types of cancer. For example, lower TMB values have been associated with a better prognosis in clear-cell carcinoma [12]. Conversely, lower TMB values have indicated worse survival in several types of cancer, including melanoma and non-small-cell lung cancer [4]. With HCC, some studies have shown decreased survival with relatively high TMB, but this connection is complicated by the low TMB range that is normally seen [8]. One study theorized that the lower survival associated with higher TMB may be due to variability in the expression of specific immune cells, such as macrophages, which can promote tumor growth, invasion, and metastasis. The authors also noted the importance of analyzing specific genetic mutations and their role in the liver immune microenvironment. To support this, their study showed that increased expression of macrophages was correlated with advanced disease. The extent to which these cells are present in the immune microenvironment is closely tied to TMB in HCC. This may explain the trend of worse prognosis that was seen in the study [13]. The case presented here, with a very high TMB, would likely support a connection of increased TMB with a worse prognosis as the patient’s cancer, though not metastatic, had progressed to the point of clinical decompensation and drove the decision to withdraw further treatment.

When considering the limitations of this case, it is important to note the unclear etiology of this patient’s HCC. Her hepatitis C and hepatitis B status were never verified, but she did not have a history of receiving treatment for either virus. The patient had no known history of illicit drug use or blood transfusions. Similarly, there was no known history of cirrhosis whether caused by alcohol abuse or any other etiology, including potential autoimmune diseases. There is an interest in connecting TMB values with HCC etiology [3]. Such information would have also been useful in determining which patient populations would be most appropriate to apply any conclusions to. Despite this, with the prevalence of HCC throughout the world, the case and its unique attributes remain significant and provide further evidence to continue studying TMB and its various roles in cancer.

## Conclusions

HCC cases with an exceptionally high TMB that progress rapidly indicate the continued need to study such markers to guide both practitioners and patients in their decision-making. Given current research, the true range of TMB remains unclear, making it difficult to stratify and appropriately evaluate areas such as response and prognosis. TMB’s association in both treating and evaluating all cancers remains an intensely studied topic due to the continuing evolution and efficacy of immunotherapy.
